# The genome sequence of the Elephant Hawk-moth, *Deilephila elpenor *(Linnaeus, 1758)

**DOI:** 10.12688/wellcomeopenres.21012.1

**Published:** 2024-03-01

**Authors:** Douglas Boyes, Peter W.H. Holland, University of Oxford and Wytham Woods Genome Acquisition Lab, Darwin Tree of Life Barcoding collective, Wellcome Sanger Institute Tree of Life Management, Samples and Laboratory team, Wellcome Sanger Institute Scientific Operations: Sequencing Operations, Wellcome Sanger Institute Tree of Life Core Informatics team, Tree of Life Core Informatics collective, Darwin Tree of Life Consortium

**Affiliations:** 1 UK Centre for Ecology & Hydrology, Wallingford, England, UK; 2 University of Oxford, Oxford, England, UK

**Keywords:** Deilephila elpenor, Elephant Hawk-moth, genome sequence, chromosomal, Lepidoptera

## Abstract

We present a genome assembly from an individual female *Deilephila elpenor* (the Elephant Hawk-moth; Arthropoda; Insecta; Lepidoptera; Sphingidae). The genome sequence is 414.1 megabases in span. Most of the assembly is scaffolded into 30 chromosomal pseudomolecules, including the Z and W sex chromosomes. The mitochondrial genome has also been assembled and is 15.37 kilobases in length. Gene annotation of this assembly on Ensembl identified 11,748 protein coding genes.

## Species taxonomy

Eukaryota; Opisthokonta; Metazoa; Eumetazoa; Bilateria; Protostomia; Ecdysozoa; Panarthropoda; Arthropoda; Mandibulata; Pancrustacea; Hexapoda; Insecta; Dicondylia; Pterygota; Neoptera; Endopterygota; Amphiesmenoptera; Lepidoptera; Glossata; Neolepidoptera; Heteroneura; Ditrysia; Obtectomera; Bombycoidea; Sphingidae; Macroglossinae; Macroglossini; *Deilephila*; *Deilephila elpenor* (Linnaeus, 1758) (NCBI:txid283834).

## Background

The Elephant Hawk-moth *Deilephila elpenor* gains its common name from the unusual appearance of its larva. The easily recognised larva grows to around 7 cm in length and is either dark brown or bright green. The first few segments behind the head are narrow and can be extended forward; with imagination, this can make the larva resemble an elephant’s trunk as it sways side-to-side. Also noticeable are four large eye-spots: two on each side of the larval body. When touched, the larva can enlarge and swell the body region where the eye-spots lie, possibly to deter potential predators ( Easterbrook, 1985). A study using artificial caterpillars made of pastry showed that eyespots and expanded anterior segments can reduce bird attack ( Hossie & Sherratt, 2013). The larva of *D. elpenor* was described and illustrated over three centuries ago by Albin (1720) who referred to it simply as ‘the elephant’. His suggestion that the larva can swim in water by turning upside down and performing contortions of its body has no modern support.

*D. elpenor* is widely distributed across the Palaearctic, from Portugal and Ireland in the west to China, Korea, Japan and the far east of Russia ( GBIF Secretariat, 2023). There are sporadic occurrences outside this range, including several clusters of records from western Canada and the northwest of the United States ( GBIF Secretariat, 2023); these are consistent with local establishment following accidental introduction. In Britain, the species is common along river valleys in southern England, including along the River Thames, Kennet and Lea valleys ( Allan, 1947), and in the last 40 years its range has expanded greatly across northern England and Scotland ( Randle *et al.*, 2019).

Larvae of *D. elpenor* feed at night on bedstraws *Galium* sp. and willowherbs including rosebay willowherb *Chamaenerion angustifolium* and hoary willowherb *Epilobium parviflorum* ( Allan, 1947). Larval development is rapid through July and August in Northern Europe, with the pupal stage overwintering. The pink and olive-green adult is frequently encountered in June and July, when it may be seen on warm nights hovering to take nectar from flowers such as honeysuckle; it is also attracted to light. Experimental studies have shown that adult *D. elpenor* can discriminate colours even in light conditions comparable to dim starlight: this research was one of the first demonstrations of nocturnal colour vision in animals ( Kelber *et al.*, 2002). Electrophysiology of motion-sensitive neurons also revealed remarkable visual sensitivity to low velocity motion, necessary for feeding by hovering at night ( Theobald *et al.*, 2010).

A genome sequence of *Deilephila elpenor* was determined as part of the Darwin Tree of Life project. The genome sequence will facilitate research into insect vision, neurobiology and host plant choice, and contribute to the growing set of resources for studying molecular evolution in the Lepidoptera.

## Genome sequence report

**Figure 1.**
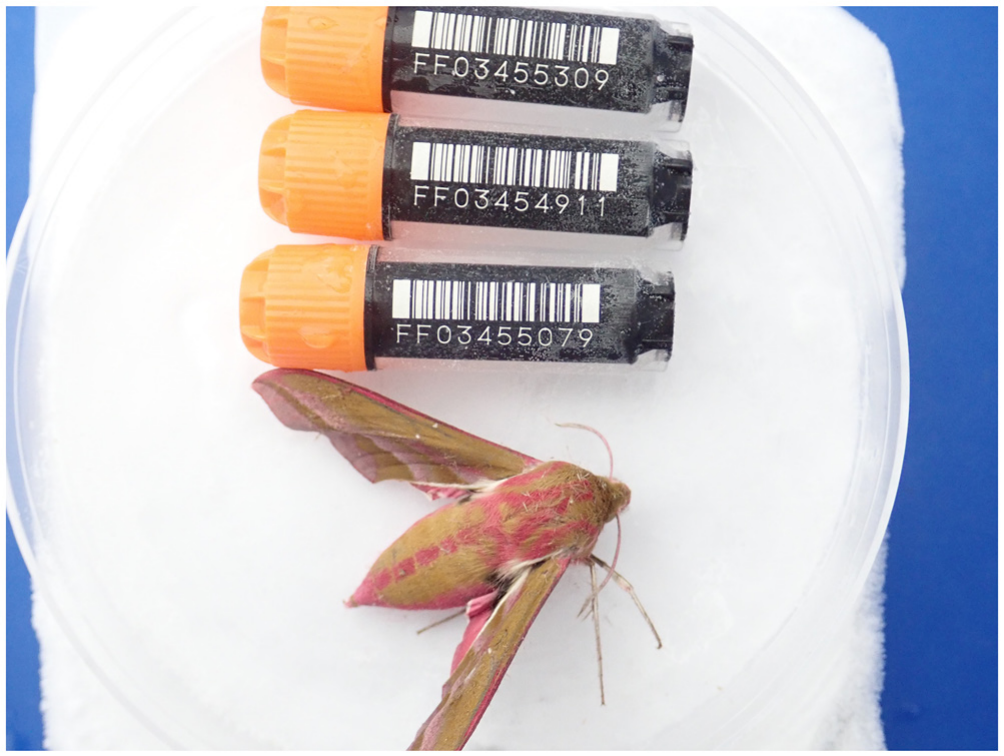
Photograph of the *Deilephila elpenor* (ilDeiElpe2) specimen used for genome sequencing.

The genome was sequenced from one female *Deilephila elpenor* ( Figure 1) collected from Wytham Woods, Oxfordshire, UK (51.77, –1.34). A total of 54-fold coverage in Pacific Biosciences single-molecule HiFi long reads was generated. Primary assembly contigs were scaffolded with chromosome conformation Hi-C data. Manual assembly curation corrected 10 missing joins or mis-joins and removed one haplotypic duplication, reducing the scaffold number by 15.79%, and increasing the scaffold N50 by 1.00%.

**Figure 2.**
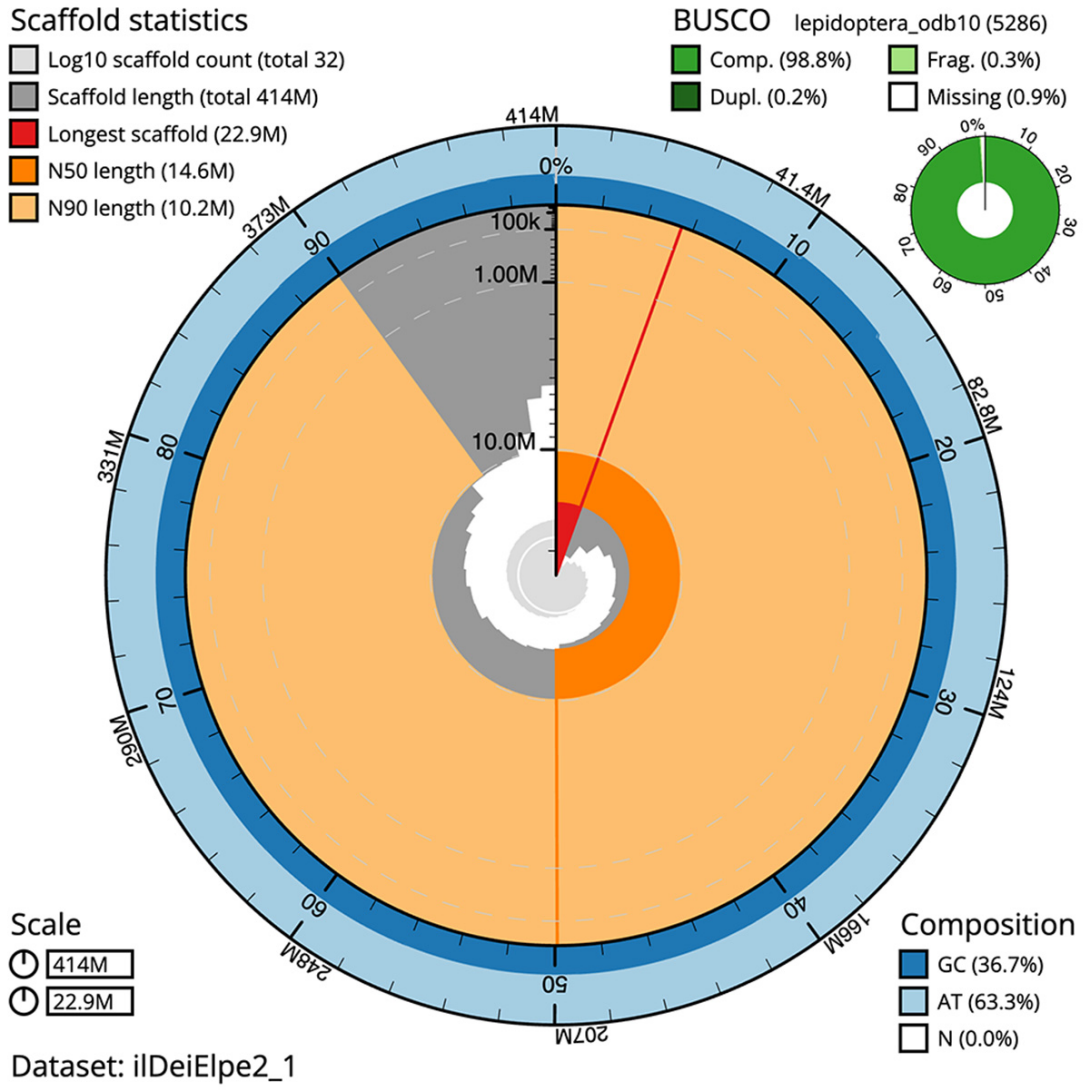
Genome assembly of *Deilephila elpenor*, ilDeiElpe2.1: metrics. The BlobToolKit Snailplot shows N50 metrics and BUSCO gene completeness. The main plot is divided into 1,000 size-ordered bins around the circumference with each bin representing 0.1% of the 414,066,290 bp assembly. The distribution of scaffold lengths is shown in dark grey with the plot radius scaled to the longest scaffold present in the assembly (22,915,455 bp, shown in red). Orange and pale-orange arcs show the N50 and N90 scaffold lengths (14,585,127 and 10,162,166 bp), respectively. The pale grey spiral shows the cumulative scaffold count on a log scale with white scale lines showing successive orders of magnitude. The blue and pale-blue area around the outside of the plot shows the distribution of GC, AT and N percentages in the same bins as the inner plot. A summary of complete, fragmented, duplicated and missing BUSCO genes in the lepidoptera_odb10 set is shown in the top right. An interactive version of this figure is available at https://blobtoolkit.genomehubs.org/view/ilDeiElpe2_1/dataset/ilDeiElpe2_1/snail.

**Figure 3.**
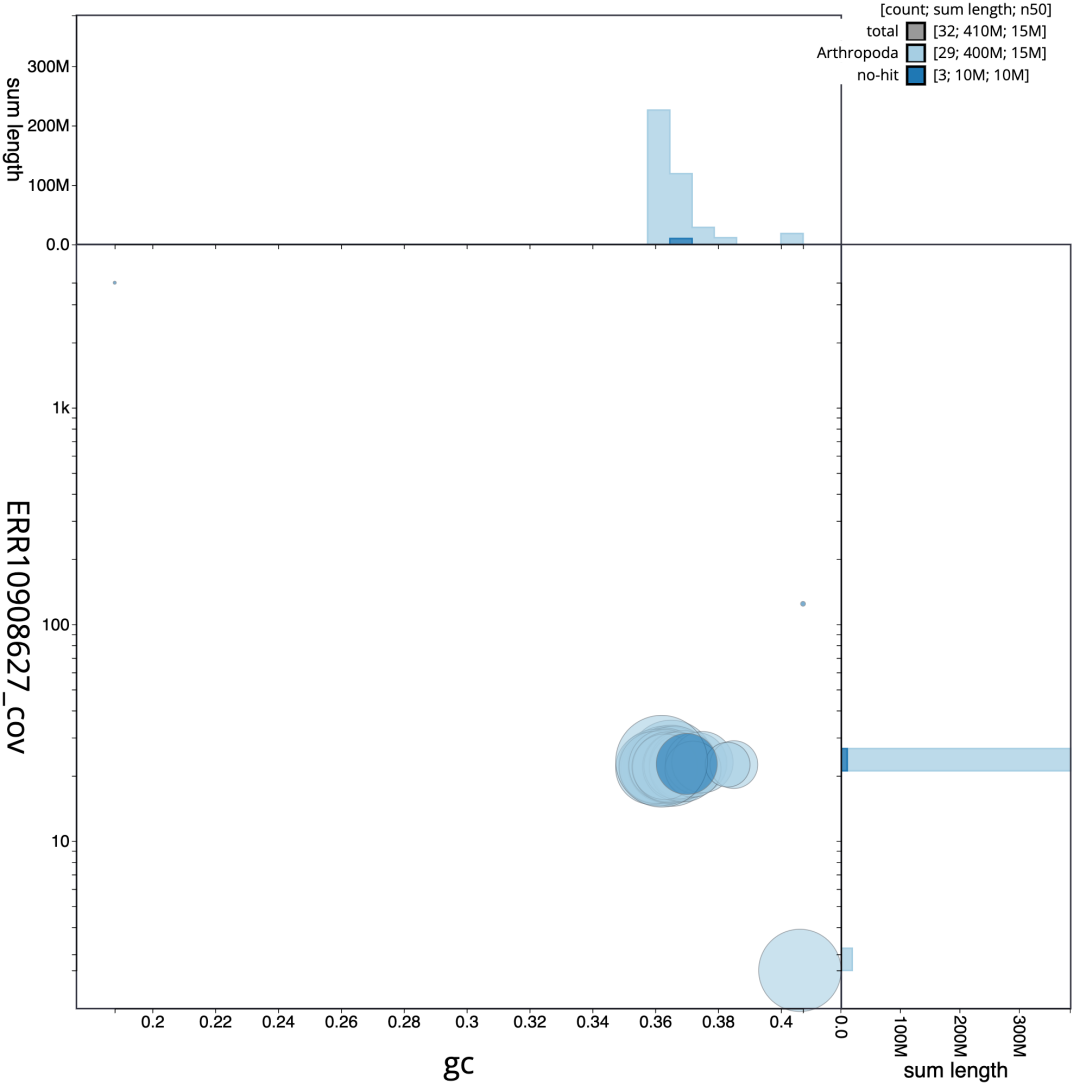
Genome assembly of *Deilephila elpenor*, ilDeiElpe2.1: BlobToolKit GC-coverage plot. Scaffolds are coloured by phylum. Circles are sized in proportion to scaffold length. Histograms show the distribution of scaffold length sum along each axis. An interactive version of this figure is available at https://blobtoolkit.genomehubs.org/view/ilDeiElpe2_1/dataset/ilDeiElpe2_1/blob.

**Figure 4.**
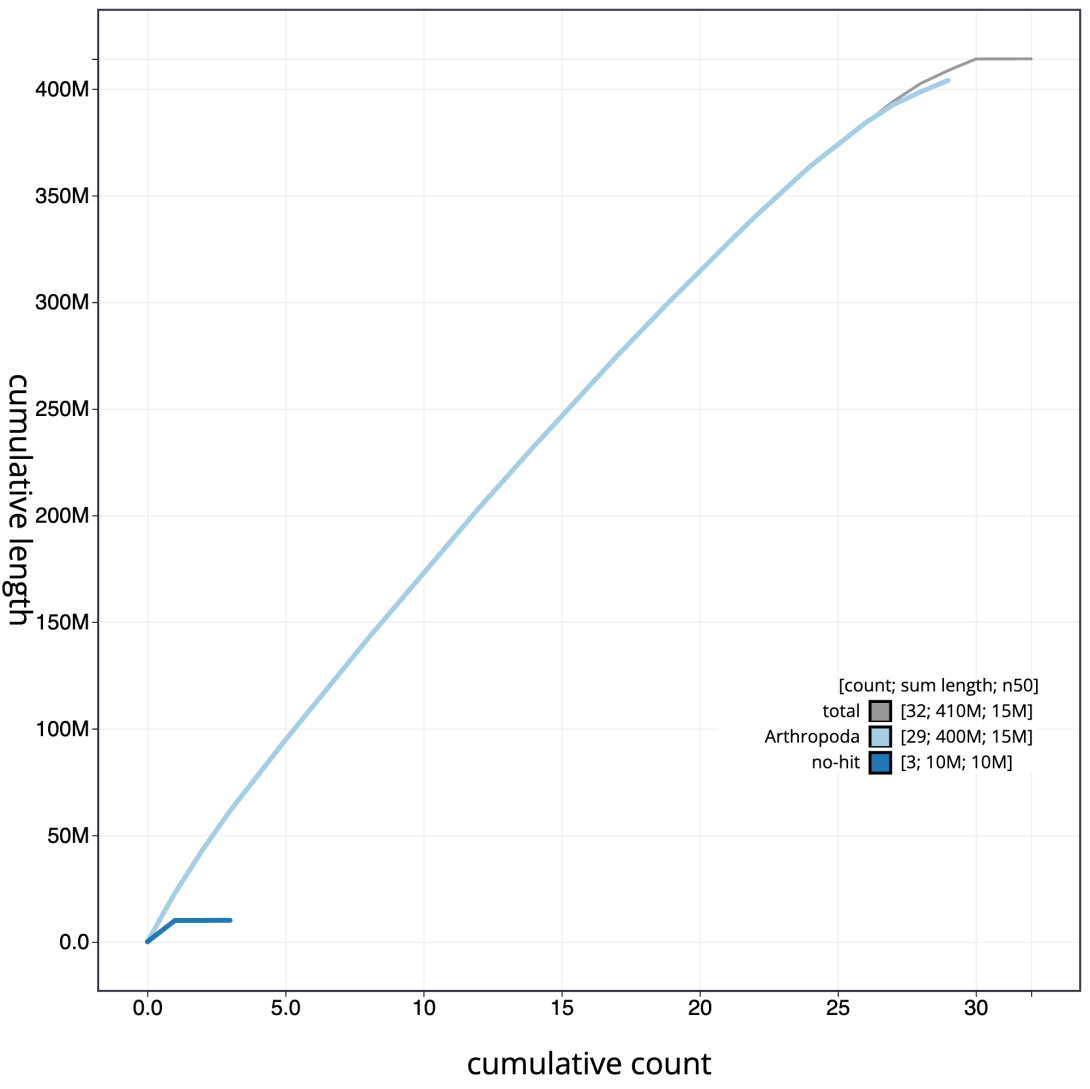
Genome assembly of *Deilephila elpenor*, ilDeiElpe2.1: BlobToolKit cumulative sequence plot. The grey line shows cumulative length for all scaffolds. Coloured lines show cumulative lengths of scaffolds assigned to each phylum using the buscogenes taxrule. An interactive version of this figure is available at https://blobtoolkit.genomehubs.org/view/ilDeiElpe2_1/dataset/ilDeiElpe2_1/cumulative.

**Figure 5.**
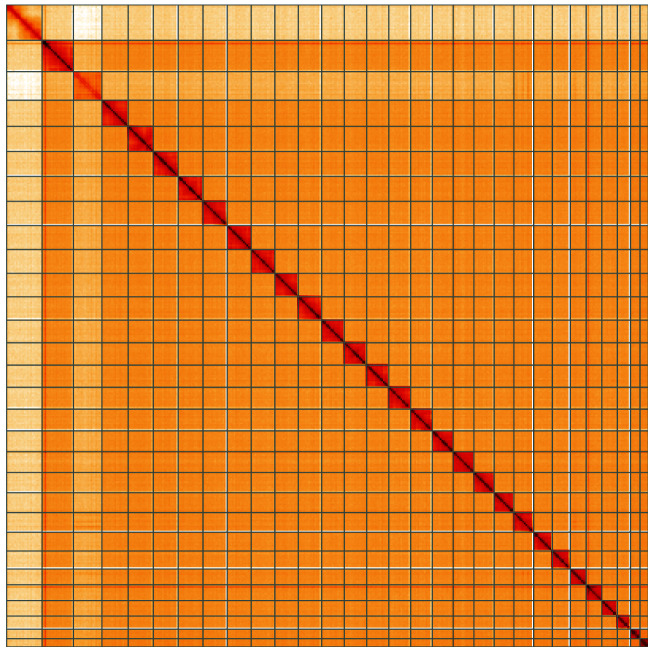
Genome assembly of *Deilephila elpenor*, ilDeiElpe2.1: Hi-C contact map of the ilDeiElpe2.1 assembly, visualised using HiGlass. Chromosomes are shown in order of size from left to right and top to bottom. An interactive version of this figure may be viewed at https://genome-note-higlass.tol.sanger.ac.uk/l/?d=f1cVHAvTSO62HOfQfV56FA.

The final assembly has a total length of 414.1 Mb in 31 sequence scaffolds with a scaffold N50 of 14.6 Mb ( Table 1). The snailplot in Figure 2 provides a summary of the assembly statistics, while the distribution of assembly scaffolds on GC proportion and coverage is shown in Figure 3. The cumulative assembly plot in Figure 4 shows curves for subsets of scaffolds assigned to different phyla. Most (99.99%) of the assembly sequence was assigned to 30 chromosomal-level scaffolds, representing 28 autosomes and the Z and W sex chromosomes. Chromosome-scale scaffolds confirmed by the Hi-C data are named in order of size ( Figure 5; Table 2). The Z chromosome identified by coverage and alignment to *Deilephila porcellus* (GCA_905220455.2) ( Boyes *et al.*, 2022). The W chromosome was identified by coverage. While not fully phased, the assembly deposited is of one haplotype. Contigs corresponding to the second haplotype have also been deposited. The mitochondrial genome was also assembled and can be found as a contig within the multifasta file of the genome submission.

**Table 1.** Genome data for *Deilephila elpenor*, ilDeiElpe2.1.

Project accession data		
Assembly identifier	ilDeiElpe2.1	
Species	*Deilephila elpenor*	
Specimen	ilDeiElpe2	
NCBI taxonomy ID	283834	
BioProject	PRJEB59948	
BioSample ID	SAMEA8603126	
Isolate information	ilDeiElpe2, female: thorax (DNA and RNA sequencing), head (Hi-C sequencing)	
Assembly metrics *		*Benchmark*
Consensus quality (QV)	66.8	*≥ 50*
*k*-mer completeness	100.0%	*≥ 95%*
BUSCO **	C:98.8%[S:98.6%,D:0.2%],F:0.3%,M:0.9%,n:5,286	*C ≥ 95%*
Percentage of assembly mapped to chromosomes	99.99%	*≥ 95%*
Sex chromosomes	ZW	*localised homologous pairs*
Organelles	Mitochondrial genome: 15.37 kb	*complete single alleles*
Raw data accessions		
PacificBiosciences SEQUEL II	ERR10906093	
Hi-C Illumina	ERR10908630	
PolyA RNA-Seq Illumina	ERR11242530	
Genome assembly		
Assembly accession	GCA_949752805.1	
*Accession of alternate haplotype*	GCA_949752825.1	
Span (Mb)	414.1	
Number of contigs	83	
Contig N50 length (Mb)	10.0	
Number of scaffolds	31	
Scaffold N50 length (Mb)	14.6	
Longest scaffold (Mb)	22.92	
Genome annotation		
Number of protein-coding genes	11,748	
Number of non-coding genes	1,790	
Number of gene transcripts	21,478	

* Assembly metric benchmarks are adapted from column VGP-2020 of “Table 1: Proposed standards and metrics for defining genome assembly quality” from ( Rhie *et al.*, 2021). ** BUSCO scores based on the lepidoptera_odb10 BUSCO set using version 5.3.2. C = complete [S = single copy, D = duplicated], F = fragmented, M = missing, n = number of orthologues in comparison. A full set of BUSCO scores is available at https://blobtoolkit.genomehubs.org/view/ilDeiElpe2_1/dataset/ilDeiElpe2_1/busco.

**Table 2.** Chromosomal pseudomolecules in the genome assembly of *Deilephila elpenor*, ilDeiElpe2

INSDC accession	Chromosome	Length (Mb)	GC%
OX457094.1	1	20.2	36.5
OX457096.1	2	16.9	36.5
OX457097.1	3	16.23	36.5
OX457098.1	4	16.04	36.5
OX457099.1	5	16.01	36.5
OX457100.1	6	15.64	36.5
OX457101.1	7	15.46	36.0
OX457102.1	8	15.27	36.0
OX457103.1	9	15.15	36.5
OX457104.1	10	15.08	36.5
OX457105.1	11	14.59	36.0
OX457106.1	12	14.44	36.0
OX457107.1	13	14.25	36.5
OX457108.1	14	14.11	36.0
OX457109.1	15	13.97	36.5
OX457110.1	16	13.43	36.5
OX457111.1	17	13.38	36.5
OX457112.1	18	13.03	36.5
OX457113.1	19	12.81	37.0
OX457114.1	20	12.69	37.0
OX457115.1	21	12.09	36.5
OX457116.1	22	11.62	36.5
OX457117.1	23	10.16	37.5
OX457118.1	24	10.14	37.5
OX457119.1	25	10.01	37.0
OX457120.1	26	8.47	37.0
OX457121.1	27	6.12	38.5
OX457122.1	28	5.38	38.5
OX457095.1	W	18.41	40.5
OX457093.1	Z	22.92	36.0
OX457123.1	MT	0.02	19.0

The estimated Quality Value (QV) of the final assembly is 66.8 with *k*-mer completeness of 100.0%, and the assembly has a BUSCO v5.3.2 completeness of 98.8% (single = 98.6%, duplicated = 0.2%), using the lepidoptera_odb10 reference set ( *n* = 5,286).

Metadata for specimens, barcode results, spectra estimates, sequencing runs, contaminants and pre-curation assembly statistics are given at https://links.tol.sanger.ac.uk/species/283834.

## Genome annotation report

The *Deilephila elpenor* genome assembly (GCA_949752805.1) was annotated at the European Bioinformatics Institute (EBI) using the Ensembl rapid annotation pipeline ( Table 1; https://rapid.ensembl.org/Deilephila_elpenor_GCA_949752805.1/Info/Index). The resulting annotation includes 21,478 transcribed mRNAs from 11,748 protein-coding and 1,790 non-coding genes.

## Methods

### Sample acquisition and nucleic acid extraction

A female *Deilephila elpenor* (specimen ID Ox000661, ToLID ilDeiElpe2) was collected from Wytham Woods, Oxfordshire (biological vice-county Berkshire), UK (latitude 51.77, longitude –1.34) on 2020-07-20 using a light trap. The specimen was collected and identified by Douglas Boyes (University of Oxford) and preserved on dry ice.

The workflow for high molecular weight (HMW) DNA extraction at the Wellcome Sanger Institute (WSI) includes a sequence of core procedures: sample preparation; sample homogenisation, DNA extraction, fragmentation, and clean-up. In sample preparation, the ilDeiElpe2 sample was weighed and dissected on dry ice ( Jay *et al.*, 2023). Tissue from the thorax was homogenised using a PowerMasher II tissue disruptor ( Denton *et al.*, 2023a). HMW DNA was extracted in the WSI Scientific Operations core using the Automated MagAttract v2 protocol ( Oatley *et al.*, 2023). The DNA was sheared into an average fragment size of 12–20 kb in a Megaruptor 3 system with speed setting 31 ( Bates *et al.*, 2023). Sheared DNA was purified by solid-phase reversible immobilisation ( Strickland *et al.*, 2023): in brief, the method employs a 1.8X ratio of AMPure PB beads to sample to eliminate shorter fragments and concentrate the DNA. The concentration of the sheared and purified DNA was assessed using a Nanodrop spectrophotometer and Qubit Fluorometer and Qubit dsDNA High Sensitivity Assay kit. Fragment size distribution was evaluated by running the sample on the FemtoPulse system.

RNA was extracted from thorax tissue of ilDeiElpe2 in the Tree of Life Laboratory at the WSI using the RNA Extraction: Automated MagMax™ *mir*Vana protocol ( do Amaral *et al.*, 2023). The RNA concentration was assessed using a Nanodrop spectrophotometer and a Qubit Fluorometer using the Qubit RNA Broad-Range Assay kit. Analysis of the integrity of the RNA was done using the Agilent RNA 6000 Pico Kit and Eukaryotic Total RNA assay.

Protocols developed by the WSI Tree of Life laboratory are publicly available on protocols.io ( Denton *et al.*, 2023b).

### Sequencing

Pacific Biosciences HiFi circular consensus DNA sequencing libraries were constructed according to the manufacturers’ instructions. Poly(A) RNA-Seq libraries were constructed using the NEB Ultra II RNA Library Prep kit. DNA and RNA sequencing was performed by the Scientific Operations core at the WSI on Pacific Biosciences SEQUEL II (HiFi) and Illumina NovaSeq 6000 (RNA-Seq) instruments. Hi-C data were also generated from head tissue of ilDeiElpe2 using the Arima2 kit and sequenced on the Illumina NovaSeq 6000 instrument.

### Genome assembly, curation and evaluation

Assembly was carried out with Hifiasm ( Cheng *et al.*, 2021) and haplotypic duplication was identified and removed with purge_dups ( Guan *et al.*, 2020). The assembly was then scaffolded with Hi-C data ( Rao *et al.*, 2014) using YaHS ( Zhou *et al.*, 2023). The assembly was checked for contamination and corrected using the TreeVal pipeline ( Pointon *et al.*, 2023). Manual curation was performed using JBrowse2 ( Diesh *et al.*, 2023), HiGlass ( Kerpedjiev *et al.*, 2018) and PretextView ( Harry, 2022). The mitochondrial genome was assembled using MitoHiFi ( Uliano-Silva *et al.*, 2023), which runs MitoFinder ( Allio *et al.*, 2020) or MITOS ( Bernt *et al.*, 2013) and uses these annotations to select the final mitochondrial contig and to ensure the general quality of the sequence.

A Hi-C map for the final assembly was produced using bwa-mem2 ( Vasimuddin *et al.*, 2019) in the Cooler file format ( Abdennur & Mirny, 2020). To assess the assembly metrics, the *k*-mer completeness and QV consensus quality values were calculated in Merqury ( Rhie *et al.*, 2020). This work was done using Nextflow ( Di Tommaso *et al.*, 2017) DSL2 pipelines “sanger-tol/readmapping” ( Surana *et al.*, 2023a) and “sanger-tol/genomenote” ( Surana *et al.*, 2023b). The genome was analysed within the BlobToolKit environment ( Challis *et al.*, 2020) and BUSCO scores ( Manni *et al.*, 2021; Simão *et al.*, 2015) were calculated.

Table 3 contains a list of relevant software tool versions and sources.

**Table 3.** Software tools: versions and sources

Software tool	Version	Source
BlobToolKit	4.2.1	https://github.com/blobtoolkit/blobtoolkit
BUSCO	5.3.2	https://gitlab.com/ezlab/busco
Hifiasm	0.16.1-r375	https://github.com/chhylp123/hifiasm
HiGlass	1.11.6	https://github.com/higlass/higlass
Merqury	MerquryFK	https://github.com/thegenemyers/MERQURY.FK
MitoHiFi	2	https://github.com/marcelauliano/MitoHiFi
PretextView	0.2	https://github.com/wtsi-hpag/PretextView
purge_dups	1.2.3	https://github.com/dfguan/purge_dups
sanger-tol/genomenote	v1.0	https://github.com/sanger-tol/genomenote
sanger-tol/readmapping	1.1.0	https://github.com/sanger-tol/readmapping/tree/1.1.0
Treeval		https://github.com/sanger-tol/treeval
YaHS	1.2a	https://github.com/c-zhou/yahs

### Genome annotation

Ensembl Genebuild ( Aken *et al.*, 2016) at the EBI was used to generate annotation for the *Deilephila elpenor* assembly (GCA_949752805.1). Annotation was created primarily through alignment of transcriptomic data to the genome, with gap filling via protein-to-genome alignments of a select set of proteins from UniProt ( UniProt Consortium, 2019).

### Wellcome Sanger Institute – Legal and Governance

The materials that have contributed to this genome note have been supplied by a Darwin Tree of Life Partner. The submission of materials by a Darwin Tree of Life Partner is subject to the **‘Darwin Tree of Life Project Sampling Code of Practice’**, which can be found in full on the Darwin Tree of Life website here. By agreeing with and signing up to the Sampling Code of Practice, the Darwin Tree of Life Partner agrees they will meet the legal and ethical requirements and standards set out within this document in respect of all samples acquired for, and supplied to, the Darwin Tree of Life Project.

Further, the Wellcome Sanger Institute employs a process whereby due diligence is carried out proportionate to the nature of the materials themselves, and the circumstances under which they have been/are to be collected and provided for use. The purpose of this is to address and mitigate any potential legal and/or ethical implications of receipt and use of the materials as part of the research project, and to ensure that in doing so we align with best practice wherever possible. The overarching areas of consideration are:

•       Ethical review of provenance and sourcing of the material

•       Legality of collection, transfer and use (national and international)

Each transfer of samples is further undertaken according to a Research Collaboration Agreement or Material Transfer Agreement entered into by the Darwin Tree of Life Partner, Genome Research Limited (operating as the Wellcome Sanger Institute), and in some circumstances other Darwin Tree of Life collaborators.

## Data Availability

European Nucleotide Archive: *Deilephila elpenor* (elephant hawk-moth). Accession number PRJEB59948; https://identifiers.org/ena.embl/PRJEB59948 ( Wellcome Sanger Institute, 2023). The genome sequence is released openly for reuse. The *Deilephila elpenor* genome sequencing initiative is part of the Darwin Tree of Life (DToL) project. All raw sequence data and the assembly have been deposited in INSDC databases. Raw data and assembly accession identifiers are reported in Table 1.

## Author information

Members of the University of Oxford and Wytham Woods Genome Acquisition Lab are listed here: https://doi.org/10.5281/zenodo.7125292.

Members of the Darwin Tree of Life Barcoding collective are listed here: https://doi.org/10.5281/zenodo.4893703.

Members of the Wellcome Sanger Institute Tree of Life Management, Samples and Laboratory team are listed here: https://doi.org/10.5281/zenodo.10066175.

Members of Wellcome Sanger Institute Scientific Operations: Sequencing Operations are listed here: https://doi.org/10.5281/zenodo.10043364.

Members of the Wellcome Sanger Institute Tree of Life Core Informatics team are listed here: https://doi.org/10.5281/zenodo.10066637.

Members of the Tree of Life Core Informatics collective are listed here: https://doi.org/10.5281/zenodo.5013541.

Members of the Darwin Tree of Life Consortium are listed here: https://doi.org/10.5281/zenodo.4783558.
