# Supplementary material for: The genome sequence of the Elephant Hawk-moth, Deilephila elpenor (Linnaeus, 1758)
Source: Wellcome Open Res. 2024 Mar 1;9:104. [Version 1] doi: 10.12688/wellcomeopenres.21012.1 (PMC11375399; doi:10.12688/wellcomeopenres.21012.1)
Supplement: Peer review history

# Reviewer report: Reviewer response for version 1

**DOI:** 10.21956/wellcomeopenres.23247.r93952

**Published:** 2024-09-04

**Authors:** Axel Künstner

**Affiliations:** 1 University of Lübeck, Lübeck, Germany

This report presents the female genome assembly of the Elephant Hawk-moth ( *Deilephila elpenor*), which spans 414.1 megabases of sequence data assembled into 30 chromosomal pseudomolecules, including the Z and W sex chromosomes. Additionally, the mitochondrial genome has also been assembled.

 The methods used are sound, software resources used are well described, and the data is available via the European Nucleotide Archive.

Are sufficient details of methods and materials provided to allow replication by others?

Yes

Is the rationale for creating the dataset(s) clearly described?

Yes

Are the datasets clearly presented in a useable and accessible format?

Yes

Are the protocols appropriate and is the work technically sound?

Yes

Reviewer Expertise:

Cancer genomics, Microbiome, Bioinformatics, Computational Biology

I confirm that I have read this submission and believe that I have an appropriate level of expertise to confirm that it is of an acceptable scientific standard.

# Reviewer report: Reviewer response for version 1

**DOI:** 10.21956/wellcomeopenres.23247.r90853

**Published:** 2024-08-16

**Authors:** Bhagya Thimmappa

**Affiliations:** 1 ORISE Postdoctoral Fellow at USDA-ARS, Chatsworth, New Jersey, USA

In the work *" **The genome sequence of the Elephant Hawk-moth, Deilephila elpenor (Linnaeus, 1758)**,"* the authors report a nuclear genome assembly using PacBio sequencing, structural annotation, and mitochondrial genome assembly.

 Comments:

 The reported assembly is nearly complete and appears to be the first genome assembly reported for *Deilephila elpenor. *A thorough functional annotation could improve its quality further as a reference genome. Additional information on genome architecture, like repeats and duplicated regions, could also provide more insights into the reported genome.

Are sufficient details of methods and materials provided to allow replication by others?

Yes

Is the rationale for creating the dataset(s) clearly described?

Yes

Are the datasets clearly presented in a useable and accessible format?

Yes

Are the protocols appropriate and is the work technically sound?

Yes

Reviewer Expertise:

Bioinformatics, Genomics, Transcriptomics, Metagenomics

I confirm that I have read this submission and believe that I have an appropriate level of expertise to confirm that it is of an acceptable scientific standard.
